# Palatable Flavoured Fluids without Carbohydrates and Electrolytes Do Not Enhance Voluntary Fluid Consumption in Male Collegiate Basketball Players in the Heat

**DOI:** 10.3390/nu13124197

**Published:** 2021-11-23

**Authors:** Bernadette Cherianne Taim, Haresh T. Suppiah, Jericho Wee, Marcus Lee, Jason K. W. Lee, Michael Chia

**Affiliations:** 1 Physical Education and Sports Science Group, National Institute of Education, Nanyang Technological University, Singapore 637616, Singapore; btaim@research.ait.ie; 2 National Youth Sports Institute, Singapore 397778, Singapore; h.suppiah@latrobe.edu.au (H.T.S.); jericho_wee@u.nus.edu (J.W.); marcus_lee@nysi.org.sg (M.L.); 3 Sport and Exercise Science, School of Allied Health, Human Services and Sport, La Trobe University, Bundoora, VIC 3086, Australia; 4 Human Potential Translational Research Programme, Yong Loo Lin School of Medicine, National University of Singapore, Singapore 119228, Singapore; phsjlkw@nus.edu.sg; 5 Department of Physiology, Yong Loo Lin School of Medicine, National University of Singapore, Singapore 117593, Singapore; 6 Global Asia Institute, National University of Singapore, Singapore 119076, Singapore; 7 N.1 Institute for Health, National University of Singapore, Singapore 117456, Singapore; 8 Institute for Digital Medicine, National University of Singapore, Singapore 117456, Singapore; 9 Singapore Institute for Clinical Sciences, Agency for Science, Technology and Research (A*STAR), Singapore 117609, Singapore

**Keywords:** small-sided games, hydration, hypohydration, dehydration, taste, team sport, high-intensity exercise performance, palatability

## Abstract

Using palatable fluids to enhance drinking in athletes who display insufficient compensatory hydration behaviour may mitigate the risks of hypohydration and performance deficits. However, it is unclear whether flavour can independently enhance fluid consumption. This study examined the effects of a colourless, artificially sweetened flavoured water (FW), without carbohydrates and with negligible amounts of sodium, compared to plain water (W) on fluid consumption in male collegiate basketball players in a practical game setting. Eighteen male basketball players (age 23.1 ± 1.3 years) played a 3v3 basketball small-sided game. The players were randomly assigned to consume either FW or W. Pre-game urine-specific gravity, fluid consumption, body mass, and hedonic taste perceptions were assessed. Basketball performance was analysed through notational analysis. Ratings of perceived exertion and thirst were recorded at pre-, post-game, and at each rest period. Heart rate was recorded throughout the gameplay. Despite significantly higher hedonic ratings for FW than W (6.78 ± 0.83 vs. 5.56 ± 1.33, *p* = 0.033, d = 1.36), there were no significant differences in fluid consumption (1083 ± 32 mL vs. 1421 ± 403 mL, *p* = 0.068, d = 0.92). Our result highlighted that using palatable fluids as a strategy to increase fluid consumption during high-intensity gameplay in the heat may not be effective if used without carbohydrates and electrolytes. Practitioners could consider both fluid palatability and composition in establishing a hydration plan for athletes.

## 1. Introduction

Optimal hydration plays a key role in the health and sport performance of athletes. While optimal fluid balance is dependent on a multitude of factors, it is conventionally defined as the avoidance of body water deficits greater than 2–3% of body mass while preventing excessive fluid consumption (i.e., overhydration) [1]. During exercise in hot-humid environments, metabolic heat produced is primarily dissipated through sweat evaporation. High sweat rates in these environmental conditions further disrupt fluid balance, and increase an athlete’s risk of hypohydration [2]. Beyond environmental conditions, other determinants, such as fluid availability, exercise intensity, and intrinsic factors (e.g., sex, acclimatisation status, thirst drive), can contribute to the risk of fluid imbalances during exercise [3].

Team sports are often characterized by intermittent bursts of high intensity work which can elicit significant sweat losses [4]. Studies showed that a fluid deficit of 2% body mass loss can compromise performance, and progressive dehydration of 3–4% body mass loss can impair cognitive function, and sport-specific technical and physical skills of team sport players [5]. Specifically, shooting performance can potentially be reduced at 2–4% body mass hypohydration [5]. This finding is crucial for basketball players, as points scored dictate the outcome of a game.

Fluid needs are highly individualistic, and achieving an optimal level of hydration for performance can be complex. Establishing a hydration plan that accounts for the individual player in the context of the environment and sport may be effective in developing strategies to optimise their hydration needs [3]. Specifically, to mitigate the risks of hypohydration and potential performance decrements, players who are identified to have high sweat rates and insufficient fluid replacement practices (e.g., reluctant drinkers) may benefit from strategies to increase fluid consumption.

Fluid palatability, defined as the hedonic evaluation of sensory factors, such as taste and smell, is a major determinant of fluid consumption [6,7]. To elaborate, palatability is associated with hedonic value (i.e., pleasantness), and leads to increased and sustained food or fluid consumption [7]. During exercise, cool and pleasant tasting drinks have been shown to enhance fluid consumption and improve fluid balance [8,9]. Studies showed that the hedonic value of fluids tends to enhance voluntary fluid consumption compared to plain water [10,11,12]. These studies utilised solutions that combine either electrolytes (i.e., sodium) and/or carbohydrates to produce flavour, and observed enhanced drinking behaviour and improved hydration status among participants. These observations can be explained by the activation of the thirst mechanism in response to sodium, as well as the behavioural pleasantness response associated with the ingestion of carbohydrates [13,14]. However, it is unclear if fluid palatability that is independent of fluid composition (i.e., carbohydrates/electrolytes) can similarly enhance voluntary fluid consumption.

Research on fluid palatability and fluid consumption in hot-humid environments has been predominantly conducted in a laboratory setting (e.g., controlled environment using a treadmill, cycle ergometer) and performed at relatively low exercise intensities [15,16,17]. Furthermore, there are limited studies that reflect how fluid consumption may be influenced by fluid palatability among team sport players in an ecologically valid and on-field setting.

The aim of this study was to examine if fluid palatability that is independent of fluid composition can enhance voluntary fluid consumption in male collegiate basketball players in a practical game setting. Though we focused primarily on examining hydration behaviour, we also sought to address the lack of information available on the physiological demands and fluid needs of small-sided games (SSG) (i.e., 3 × 3 basketball instead of the traditional 5 × 5). Research on 3 × 3 basketball remains scarce despite its growing popularity, which culminated in its inclusion in the 2020 Tokyo Olympics, as well as the increased use of SSG in light of the COVID-19 outbreak [18,19,20]. Therefore, the objective of this study was twofold: (i) to investigate the effects of a colourless, artificially sweetened flavoured water, without carbohydrates and with negligible amounts of sodium, compared to plain water on voluntary fluid consumption and fluid balance; and (ii) to examine the influence of hydration status on physiological responses and basketball performance indicators using notational analysis.

## 2. Materials and Methods

### 2.1. Participants

Eighteen male basketball players were recruited via convenience sampling from a national varsity league in Singapore. All players were tropical natives who lived and trained in a hot and humid country. Players presented with no injury and/or illness, and were excluded from the study if they reported to be on any form of medication and/or had consumed caffeine, alcohol, or diuretics prior to the test session. Institutional ethical approval for the study was obtained (IRB-2019-06-041-01). Demographic data are in Table 1.

### 2.2. Design

We adopted a randomised between-subject design. The small-sided basketball game involved six players (3v3) on a full-court with an active playing time of 30 min. Prior to gameplay, the players were randomly assigned to consume either a colourless, flavoured water (without carbohydrates, and with negligible amounts of sodium) (FW) or plain water (W). Hydration status and hedonic taste perceptions were assessed prior to and after gameplay. Fluid consumption was determined post-game. Heart rate responses and rating of perceived exertion (RPE) were measured to assess the physiological load. Basketball performance indicators were assessed by notational analysis.

### 2.3. Procedure

#### 2.3.1. Environmental Conditions

Environmental temperature and humidity at the start of each SSG was measured using a calibrated handheld environmental meter (Kestrel 5000, Nielsen-Kellerman, Boothwyn, PA, USA). Mean environmental temperature and humidity was 31.7 ± 0.5 °C, 62 ± 4%.

#### 2.3.2. 3 × 3 Small-Sided Basketball Game

**Figure 1:**
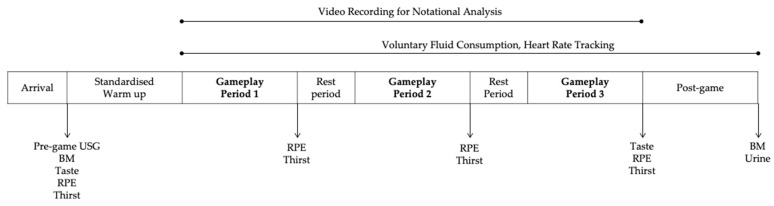
Overview of the game structure and measurements. Abbreviations: USG, urine-specific gravity; BM, body mass; RPE, ratings of perceived exertion.

Prior to gameplay, all players performed a standardised warm up protocol. The structure of the SSG involved six players (3v3) on a full-court. The total duration of the gameplay was 40 min, which consisted of three periods of 10-min active gameplay interspersed by two 5-min rest periods, where players returned to their designated rest area. Each 10-min period of active gameplay involved two 5-min halves, with a 30-s rest at half-time for the teams to swap ends of the field of play. The gameplay was officiated by a referee. The overview of the game structure is displayed in Figure 1.

This study used an SSG instead of a traditional 5 × 5 or 3 × 3 basketball game, as SSG are commonly used in basketball training to build up physical fitness and improve technical skills [23,24]. Specifically, evidence suggests that increasing playing area while reducing the number of players (e.g., 3 × 3 on full-court) can effectively increase physiological load [25,26].

#### 2.3.3. Fluid Palatability

##### Fluid Provision

Each player was randomly assigned either the FW or W condition, and was provided with two 1500 mL bottles containing their assigned fluid. Each bottle was weighed to the nearest ±0.01 kg on a digital scale (model ICS241; Mettler Toledo Pte. Ltd., Columbus, OH, USA). Both fluids were commercially available and packaged in similar transparent bottles. The players were blinded to the brand identity of the fluids. The FW was lemon-flavoured, artificially sweetened with acesulfame K and sucralose, and contained negligible amounts of sodium (<10 mg per 250 mL). Both acesulfame K and sucralose can improve fluid palatability, as they enhance sweetness and mask unpleasant bitter and sour tastes, respectively [27]. As cooler fluids increase palatability [8], the bottles were placed in an ice bath with the temperature maintained at 4.5 ± 0.8 °C throughout gameplay.

##### Palatability Ratings

A sensory questionnaire was used to measure the perceived intensities of sweetness, saltiness, and sourness [28]. Each sense of taste was rated on a 7-point category scale which ranged from “not sweet/salty/sour at all” to “very very sweet/salty/sour”. A 9-point hedonic category scale was used to determine beverage acceptability, and ranged from “dislike extremely” to “like extremely” [29]. These palatability ratings were measured ten minutes before and immediately after gameplay.

#### 2.3.4. Fluid Balance

##### Hydration Status

Pre-game hydration status was assessed on urine-specific gravity (USG) upon arrival using a clinical refractometer (model UG-alpha, ATAGO Co., Tokyo, Japan). Two USG thresholds were used to determine hypohydration: (i) USG > 1.020 and (ii) USG > 1.025. As the conventional limit of 1.020 has been shown to incorrectly classify athletes as hypohydrated when their blood serum osmolality indicates euhydration (i.e., USG false positive), a secondary threshold of 1.025 was included to increase the specificity of USG to detect hypohydration [30].

Post-game hydration status was assessed through percentage changes in body mass. Voided body mass was measured at pre- and post-game, where players towel-dried and wore their undergarments only. Body mass was measured to the nearest 0.01 kg on a digital scale (model SECA 874, SECA, Hamburg, Germany).

##### Fluid Consumption

Fluid consumption was determined from the difference between the initial mass of the bottle and the final mass of the bottle post-game. Bottle mass was measured to the nearest ±0.01 kg using a digital scale (model ICS241; Mettler Toledo Pte. Ltd., Columbus, OH, USA). The players were allowed ad libitum drinking throughout gameplay, and no attempt was made to influence the drinking behaviours of the players. The total fluid consumption for each player was calculated using the following formula [31]:

Fluid consumption (mL) = Pre-game bottle weight (g) − Post-game bottle weight (g)

##### Sweat Rate

To calculate sweat rate, urine output was collected and weighed. Though the players were specifically told that they could visit the toilet at any point throughout gameplay, none of them did so. Post-game urine output was collected and weighed. The total testing duration for all the players was two hours. The sweat rate for each player was calculated using the following formula [1]:

Body mass change (g) = Pre-game body mass (g) − Post-game body mass (g)

Sweat loss (mL) = Body mass change (g) + Fluid consumption (mL) − Urine output (g)

Sweat Rate (mL/h) = Sweat loss (mL)/2 (h)

#### 2.3.5. Subjective Measures

##### Ratings of Perceived Exertion

Ratings of perceived exertion (RPE) was recorded at pre-game, during gameplay (10- and 25-min mark), and at post-game (40-min mark) [32].

##### Thirst

Thirst was evaluated concurrently at pre-game, during gameplay (at 10- and 25-min mark), and at post-game (40-min mark) using a 9-point scale [33].

#### 2.3.6. Performance

##### Heart Rate Tracking

All players were fitted with a heart rate monitor (Firstbeat Technologies Oy, Jyväskylä, Finland), and heart rate was monitored throughout the session.

##### Notational Analysis

To assess basketball performance, statistics for the number of assists (AST), defensive rebounds (DR), and two-point and three-point field goal percentages (2P%; 3P%) were recorded through notational analysis [34]. These performance indicators effectively discriminate winning and losing teams in basketball [34]. Gameplay was recorded using the GoPro Hero 7 Black (GoPro, Inc., San Mateo, CA, USA) with image processing at 60 frames per second.

### 2.4. Statistical Analysis

Data analysis was conducted using SPSS Statistics, version 23 (IBM, Armonk, NY, USA), with statistical significance set at *p* < 0.05 with 95% confidence intervals. Results are expressed as mean ± SD or frequencies, unless otherwise stated. The independent samples *t*-test was performed to examine significant between-group differences. Effect sizes were computed, and thresholds were set at 0.2—small, 0.5—medium, and 0.8—large [35]. Where there is a trend towards statistical significance and a large effect size, the magnitude-based decisions (MBD) approach was used to assess practical significance. The differences were further evaluated on their magnitudes using standardised changes of the following scales: <0.2, trivial; 0.2 to 0.6, small; 0.6 to 1.2, moderate; 1.2 to 2.0, large; and >2.0, very large. Decisions about magnitudes accounting for the uncertainty were based on a reference-Bayesian analysis [36], which provided estimates of chances that the true magnitude was a substantial decrease or negative value, a trivial value, and a substantial increase or positive value. Clear effects are reported with a qualitative descriptor for the magnitudes with chances that are >25% using the following scale: >0.25, possibly; >0.75, likely; >0.95, very likely; >0.995, most likely [37]. When the chances of a substantial or trivial magnitude were >95%, the magnitude itself is described as clear. Effects with inadequate precision are described as unclear.

## 3. Results

At baseline, there were no differences between the pre-game USG of both groups (*p* = 0.170). The demographic information and pre-game hydration status of the players are displayed in Table 1.

**Figure 2:**
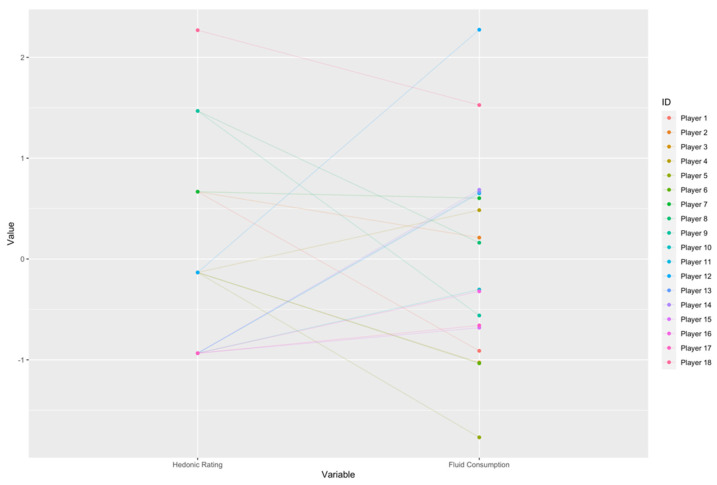
Standardised values of each individual player’s hedonic rating and fluid consumption.

Hedonic ratings were significantly higher for FW (6.78 ± 0.83) than W (5.56 ± 1.33) (*p* = 0.033). However, there were no significant differences in fluid consumption (*p* = 0.068) and body mass changes (*p* = 0.077). MBD analysis highlighted that fluid consumption was likely higher in W compared to FW, with likely lower body mass losses in FW (−0.941 ± 0.524) compared to W (−0.534 ± 0.376). The palatability ratings and hydration status are shown in Table 2. The standardised values of each individual player’s hedonic rating and corresponding fluid consumption are displayed in Figure 2.

There were no significant differences in post-game RPE, average heart rate, and basketball performance indicators (Table 3). The average heart rate reported was 176 ± 13 bpm.

## 4. Discussion

The primary objective of the study was to investigate the effects of a colourless, flavoured water, without carbohydrates and containing negligible amounts of sodium, compared to plain water on voluntary fluid consumption and fluid balance among male basketball players. The secondary objective was to examine the influence of hydration status of players on basketball performance indicators through notational analysis. The results demonstrated that fluid palatability, without added carbohydrates and/or electrolytes, do not induce any appreciable increase in voluntary fluid consumption compared to plain water. Interestingly, the higher hedonic value accorded to FW was not reflected in the players’ drinking behaviours.

To the authors’ knowledge, there have been limited studies examining the effects of fluid palatability, without added carbohydrates and electrolytes, on fluid consumption in a practical, on-field setting in the heat. In the present study, there were no significant differences in fluid consumption between both conditions. Deming et al. [38] reported similar findings, concluding that during moderate intensity, aerobic exercise in the heat, flavour does not enhance fluid consumption or fluid balance. Though their study was conducted under controlled laboratory conditions, both studies used fluids which did not contain carbohydrates and had negligible amounts of sodium. Conversely, our results contradict previous findings which reported that flavoured water enhances fluid consumption [10,11,12,39]. In a previous study by Minehan et al. [10] which examined the effect of drink flavour on fluid balance in team sport players during training, better fluid balance was achieved using flavoured drinks. Our results may conflict with those of Minehan and colleagues [10], plausibly due to differences in drink composition between studies, as the flavoured drinks in the cited study contained 18.7 mmol/L of sodium, compared to negligible amounts of sodium in the drink used in the present study.

Moreover, the conflicting findings may be explained by the intrinsic thirst response to acute sodium ingestion. Plasma osmolality is primarily a function of plasma sodium concentration in the blood, and a slight elevation (2–3%) in plasma osmolality activates the osmotic thirst mechanism [40]. Hence, sodium ingestion increases the intravascular sodium concentration, inducing thirst and voluntary fluid consumption. Furthermore, there is some evidence that carbohydrates (e.g., sucrose), but not artificial sweeteners (e.g., sucralose), activate the dopaminergic midbrain areas associated with rewards and pleasure [13]. Hence, the behavioural pleasantness response elicited by carbohydrate ingestion may enhance voluntary drinking. However, this is speculative, and further research is warranted. To that end, using palatable fluids as a strategy to increase voluntary fluid consumption during exercise in the heat may not be effective if used without carbohydrates and electrolytes.

An interesting observation in this study is the non-corresponding findings between hedonic value and fluid consumption. Despite the significantly higher hedonic values reported for FW, fluid consumption was likely higher in W. Consequently, the players who consumed W experienced slightly lower levels of dehydration. Though the underlying mechanisms behind this were unclear, there was a possibility that FW may decrease voluntary drinking. From an applied perspective, the non-corresponding findings may indicate that in addition to assessing athletes’ fluid preferences, it may also be meaningful to observe their on-field drinking behaviours.

In the present study, hydration status did not influence physiological responses and basketball performance as assessed by notational analysis. Though the players who consumed W experienced slightly lower levels of dehydration, there were no differences in physiological responses or basketball performance between both conditions. All the players appropriately gauged fluid consumption throughout gameplay, and body mass changes were within the 2% threshold. This self-regulatory drinking behaviour paralleled that of studies involving either tropical natives or heat-acclimated participants [16]. Further, a significant positive relationship was observed between fluid consumption and estimated sweat loss in a study involving tropical native football players [41]. These findings present a possibility that tropical natives may be equipped with a more reliable perception of sweat loss to guide fluid replacement.

Though we focused primarily on examining hydration behaviour, the study also provided some insights into the physiological characteristics of SSG. The structure of the SSG performed in this study involved six players (3v3) on a full-court with both baskets in play. The duration of active gameplay was shorter in the SSG compared to traditional 5v5 basketball. Heart rate data showed that the players spent an average of 44.9% of on-court playing time in the high-intensity zone (≥90% maximal heart rate) [42]. The average maximal heart rate was 189 ± 12 bpm, and the average heart rate was 176 ± 13 bpm. Despite a shorter duration of gameplay, the absolute heart rate response elicited during the SSG was comparable to that of traditional basketball [43]. Hence, it is plausible that the relative intensity of SSG can be higher than that of traditional basketball. This observation reinforces the use of SSG, specifically the manipulation of relative playing area and/or number of players on court, to elicit high physiological loads during training amidst other training benefits [24,26,44].

## 5. Limitations

The findings of this study must be interpreted in light of some limitations. The study adopted a between-group study design with a small sample size. Future studies could use a larger sample size and/or adopt a within-group study design.

Another methodological limitation was that the acceptance of artificial sweeteners was not assessed in the sensory questionnaire. Mouthfeel sensations, unlike the sensation of tastes (i.e., sweet, salty, sour), are “tactile, irritant and thermal” sensations [45]. Most high-intensity sweeteners, such as those used in the current study, do not provide the same mouthfeel as carbohydrates-based sweeteners [46]. These differences contribute to, but are not sufficiently captured in, the perceived hedonic value of foods/fluids. Though more research is needed, some anecdotal evidence suggests that artificial sweeteners trigger gastrointestinal (GI) symptoms [47]. Furthermore, health-conscious individuals may have an aversion to artificial sweeteners [48]. Therefore, future research could account for mouthfeel, GI comfort, and the acceptability of artificial sweeteners as factors that may influence perceived palatability and drinking behaviour.

Lastly, the present study only included male basketball players. Due to physiological sex differences (e.g., endocrinology), findings of this study could not be extended to female players. Future studies could consider examining sex differences in fluid preferences.

## 6. Conclusions

This study highlights that fluid palatability, without added carbohydrates and/or electrolytes, does not induce any appreciable increase in fluid consumption compared to plain water in male collegiate basketball players during a practical game setting in the heat. In comparison to previous studies, it is plausible that electrolyte and carbohydrates concentrations might play a larger role than flavour in determining fluid consumption. Hence, practitioners could consider both fluid palatability and fluid composition in establishing a hydration plan for athletes. Our results also suggested the possibility that athletes’ on-field drinking behaviours may differ from their reported hedonic values. From an applied perspective, it may be meaningful to observe how athletes’ reported fluid preferences translate into their on-field hydration practices.

## Figures and Tables

**Table 1:** Participant demographic characteristics and hydration values.

		Demographic Data ^1^		USG Values	Pre-Game Hydration Classification ^2^			
					Conventional USG Threshold ^3^		Secondary USG Threshold ^4^	
	*N*	Age (years)	Mass (kg)	Pre-Game USG	Euhydrated (≤1.020)	Hypohydrated (>1.020)	Euhydrated (≤1.025)	Hypohydrated (>1.025)
FW	9	23.4 (1.4)	76.4 (9.0)	1.021 (0.007)	3 (33.3)	6 (66.7)	6 (66.7)	3 (33.3)
W	9	22.8 (1.2)	76.6 (15.1)	1.016 (0.009)	5 (55.6)	4 (44.4)	8 (88.9)	1 (11.1)
Overall	18	23.1 (1.3)	76.5 (12.1)	1.018 (0.008)	8 (44.4)	10 (55.6)	14 (77.8)	4 (22.2)

Abbreviations: FW = flavoured water; W = plain water; USG = urine-specific gravity. Note: ^1^ Data expressed as mean (SD); ^2^ Data presented as *n* (%), where *n* = number of observations; ^3^ Classifications based on Cheuvront et al. [21]; euhydrated ≤ 1.020; hypohydrated > 1.020; ^4^ Classifications based on recommendations to raise hypohydration classification thresholds to increase specificity [22]; euhydrated ≤ 1.025; hypohydrated > 1.025.

**Table 2:** Between-group differences in palatability ratings and hydration status.

		Mean (SD)		Mean Difference (95% CI)	*p* Value	Effect Size (95% CI)	MBD
		FW	W				
Palatability Ratings	Hedonic Rating	6.78 (0.83)	5.56 (1.33)	1.22 (0.11 to 2.33)	0.033	1.10 (0.01 to 2.14)	
	Sweetness	4.44 (1.42)	2.00 (1.50)	2.44 (0.98 to 3.91)	0.003	1.67 (0.418 to 2.87)	
	Saltiness	3.89 (0.78)	2.11 (1.45)	1.78 (0.61 to 2.94)	0.007	1.52 (0.32 to 2.68)	
	Sourness	4.33 (1.32)	2.22 (1.39)	2.11 (0.75 to 3.47)	0.005	1.55 (0.34 to 2.71)	
Hydration Status	Fluid Consumption (mL)	1083 (326)	1421 (403)	−338 (−705 to 28.6)	0.068	−0.92 * (−1.92 to 0.13)	Likely; Moderate
	Sweat Rate (mL/h)	865 (117)	874 (165)	−9.06 (−152 to 134)	0.895	−0.06 (−0.99 to 0.86)	
	Change in BM (%)	−0.93 (0.52)	−0.53 (0.37)	−0.40 (−0.85 to 0.05)	0.077	−0.89 * (−1.89 to 0.15)	Likely; Moderate

Abbreviations: FW = flavoured water; W = water; CI = confidence interval, MBD = magnitude-based decisions; BM = body mass. Note: * MBD only reported where there is a trend towards statistical significance and a large effect size; MBD qualitative descriptor: >0.25, possibly; >0.75, likely; >0.95, very likely; >0.995, most likely; effects with inadequate precision are described as unclear. Magnitude of differences: <0.2, trivial; 0.2 to 0.6, small; 0.6 to 1.2, moderate; 1.2 to 2.0, large; and >2.0, very large.

**Table 3:** Between-group differences in physiological measures and basketball performance parameters.

		Mean (SD)		Mean Difference (95% CI)	*p* Value	Effect Size (95% CI)
		FW	W			
Physiological Measures	RPE	5.78 (1.56)	6.56 (1.88)	−0.78 (−2.50 to 0.95)	0.354	−0.45 (−1.39 to 0.51)
	Average heart rate (bpm)	172 (12)	179 (14)	−7 (−19 to 6)	0.289	−0.52 (−1.46 to 0.46)
Basketball Performance	2P%	49.20 (12.00)	51.89 (7.75)	−2.64 (−12.77 to 7.48)	0.587	−0.26 (−1.19 to 0.68)
	3P%	17.67 (16.73)	9.79 (18.66)	7.88 (−9.83 to 25.58)	0.360	0.44 (−0.15 to 1.38)
	AST	6.11 (3.26)	6.78 (3.07)	−0.67 (−3.83 to 2.50)	0.661	−0.47 (−1.41 to 0.49)
	DR	7.00 (4.06)	7.67 (3.64)	−0.67 (−4.52 to 3.19)	0.719	−0.03 (−0.95 to 0.89)

Abbreviations: RPE = ratings of perceived exertion, FW = flavoured water; W = unflavoured water; CI, confidence interval; 2P% = 2-point field-goal percentage; 3P% = 3-point field-goal percentage; AST = number of assists; DR = number of defensive rebounds.

## Data Availability

The data presented in this study are available on request from the corresponding author. The data are not publicly available due to privacy and ethical issues.
